# Metabolome profiling across liver lobes and metabolic shifts of the MASLD mice

**DOI:** 10.1186/s12263-025-00768-7

**Published:** 2025-04-16

**Authors:** Xiaolin Ma, Wenbo Bian, Wenting Song, Yitong Lu, Zhen Wang, Zhenyu Yao, Qiuhui Xuan

**Affiliations:** 1 Department of Endocrinology, Shandong Provincial Hospital, Shandong University; Key Laboratory of Endocrine Glucose & Lipids Metabolism and Brain Aging, Ministry of Education, Jinan, Shandong 250021 China; 2 Shandong Key Laboratory of Endocrinology and Lipid Metabolism, Jinan, Shandong 250021 China; 3 Shandong Institute of Endocrine and Metabolic Diseases, Jinan, Shandong 250021 China; 4 “Chuangxin China” Innovation Base of Stem Cell and Gene Therapy for Endocrine Metabolic Diseases, Jinan, Shandong China; 5 Shandong Engineering Laboratory of Prevention and Control for Endocrine and Metabolic Diseases, Jinan, Shandong 250021 China; 6 Shandong Engineering Research Center of Stem Cell and Gene Therapy for Endocrine and Metabolic Diseases, Jinan, Shandong 250021 China

**Keywords:** Liver lobes, MASLD, Metabolites, Amino acid metabolism, Lipid metabolism

## Abstract

**Background:**

The mammalian liver executes its vital functions through intricate hepatic biochemistry. However, the complexity of the liver metabolome and its dynamic alterations during metabolic dysfunction-associated steatotic liver disease (MASLD) remain poorly understood.

**Methods:**

We established progressive MASLD mouse models through high-fat diet (HFD) and high-fat/high-cholesterol (HFHC) dietary-feeding across multiple time points. Utilizing liquid chromatography-mass spectrometry (LC-MS)-based metabolomics and lipidomics, we systematically mapped the metabolome atlas of the mouse liver across five anatomical segments during the progression of MASLD.

**Results:**

By integration of data from two assays, we structurally annotated 426 lipids and 118 polar metabolites. The temporal progression of HFD feeding (0, 8, and 16 weeks) resulted in gradual metabolic deterioration across various liver segments. In HFHC-fed mice, metabolic alterations surged sharply from 0 to 8 weeks, followed by moderate progression until 16 weeks in different liver segments. Elevated levels of glycerolipids and cholesteryl esters, along with fluctuating acylcarnitine and fatty acid levels across various liver segments, suggested impaired energy metabolism and disrupted fatty acid oxidation. As MASLD progresses, a shift in sphingolipid metabolism, linked to inflammation, was observed, accompanied by significant alterations in phospholipid turnover patterns. Additionally, amino acid profiles in the livers of HFD-fed and HFHC-fed mice were altered, potentially influencing the regulation of energy metabolism, inflammation, and oxidative stress. These metabolic changes in lipids and amino acids displayed segment-specific patterns, indicating varying sensitivities to inflammation and mitochondrial β-oxidation across different liver lobes. Notably, the left lateral lobe showed heightened sensitivity to metabolic disturbances during MASLD progression.

**Conclusion:**

Our findings provided in-depth understanding in hepatic metabolites of MASLD, offering a comprehensive resource for further investigation.

**Supplementary Information:**

The online version contains supplementary material available at 10.1186/s12263-025-00768-7.

## Introduction

Metabolic dysfunction-associated steatotic liver disease (MASLD) is one of the most prevalent chronic liver diseases, affecting approximately 30% of the global adult population [1–3]. Formerly known as non-alcoholic fatty liver disease (NAFLD), it has been recently reclassified as MASLD following a global multi-society consensus [4]. The disease is characterized by the excessive lipid accumulation in hepatocytes, leading to liver injury and progression to metabolic dysfunction-associated steatohepatitis (MASH) and cirrhosis [5, 6]. The increasing incidence of MASLD is closely linked to the rising prevalence of obesity, type 2 diabetes, and metabolic syndrome, positioning it as a major global health concern. In addition to liver-related complications, MASLD is associated with an elevated risk of hepatocellular carcinoma and cardiovascular diseases, further amplifying its burden on public health.

While the overall burden of MASLD is well recognized, the molecular mechanisms underlying its initiation and progression remain incompletely understood. Emerging evidence suggests that the liver is not a metabolically uniform organ; rather, distinct lobes of the liver may exhibit significant metabolic heterogeneity [7, 8]. This lobar variation in metabolic activity could play a crucial role in the disease’s development, as different regions of the liver may respond variably to lipid accumulation, inflammation, and other stressors. Such regional disparities are shaped by factors including hepatic blood flow, oxygenation gradients, and differential gene and protein expression across liver lobes. From a genetic and proteomic perspective, recent studies have revealed compelling lobar-specific variations in the expression of certain genes and proteins. For instance, genes involved in lipid metabolism, oxidative stress response, and immune regulation have showed differential expression between the central and peripheral regions of the liver lobes, as well as between the left and right lobes [9–12]. Enzymes such as fatty acid synthase (FASN) and acetyl-CoA carboxylase (ACC), as central players in lipid metabolism, have been found to be more abundantly expressed in specific liver regions, contributing to the uneven lipid distribution and the progression of MASLD [13–16]. These differences suggest that certain hepatic regions may be more vulnerable to lipid accumulation or inflammatory damage due to localized molecular profiles.

Despite these advances in characterizing hepatic heterogeneity at the genomic and proteomic levels, the precise role of these lobar variations in the development and progression of MASLD remains poorly characterized. Metabolites and lipids, as important constituents of cellular components and signaling molecules, play a pivotal role in the pathophysiology of MASLD [17]. To achieve a comprehensive understanding and effective modeling of fatty liver disease, the present study systematically sampled liver tissues from five anatomical segments of mice subjected to two widely used dietary models of MASLD: high-fat diet (HFD) and high-fat/high-cholesterol diet (HFHC). These models were applied over different durations (8 and 16 weeks) to capture the temporal dynamics of disease progression.

In this study, we applied lipid chromatography-mass spectrometry (LC-MS)-based metabolomics and lipidomics, analyzing 544 structurally annotated metabolites, to elucidate the metabolic remodeling among multiple liver lobes in the pathogenesis and progression of steatotic liver disease. This study revealed significant remodeling of lipid and amino acid metabolism in response to a MASLD-inducing diet. Furthermore, our findings uncovered distinct differences in amino acid metabolism, inflammatory response, and β-oxidation among hepatic lobes at early stage of MASH. By constructing a metabolome atlas for the MASLD mouse, encompassing multiple liver lobes, we aimed to comprehensively characterize dynamic metabolome alterations linked to hepatic steatosis. This work provided a foundational framework for future research and therapeutic strategies.

## Methods

### Methods of model construction

The animal experimental protocols and procedures were reviewed and approved by the Animal Ethics Committee of Shandong Provincial Hospital. The relevant ethical approval documents have been uploaded in the related files.

C57BL/6J mice (male, 7-week-old) were purchased from Vital River Laboratory Animal Technology and were maintained on a 12-h light/12-h dark cycle in a controlled environment at 22 ± 0.5 °C with 50–60% humidity. Food and water were provided ad libitum. Following a one-week acclimation period, mice were randomly divided into several groups based on body weight, with 10 mice per group.

For the simple fatty liver mouse models, 8-week-old male C57BL/6J mice were fed a HFD containing 60% fat from lard and soybean oil, 26% carbohydrate, and 14% protein (TP 23400; TrophicDiet, Nantong, China) for either 8 or 16 weeks [18]. For the MASH mouse models, 8-week-old male C57BL/6J mice were fed a HFHC diet consisting of 42% fat from milkfat, 44% carbohydrates, 14% protein and 0.2% cholesterol (TP 26304; TrophicDiet, Nantong, China) for either 8 or 16 weeks [19]. Detailed information on the diets were provided in Tables 1 and 2. Control mice were fed a low-fat diet (LFD) (TrophicDiet, Nantong, China) for the same durations of 8 or 16 weeks [20].

**Table 1:** Detailed composition of mouse high-fat diet

Product	HFD (TP23400)
Ingredients	g/kg
Protein (Casein, L-Cystine)	201
Carbohydrate (dextrin, sucrose)	320
Fat (soybean oil, lard)	341
Fiber (Cellulose)	71
Mineral/Vitamin/choline mixture	67
Antioxidant (TBHQ)	0.067
Total	1000
Energy, kcal/g	5.1
% kcal from Protein	14%
Carbohydrate	26%
Fat	60%
Total	100%

**Table 2:** Detailed composition of mouse high-fat/high-cholesterol diet

Product	HFHC (TP26304)
Ingredients	g/kg
Protein (Casein, L-Cystine)	178
Carbohydrate (starch, sucrose)	489
Fat (milkfat)	209
Fiber (Cellulose)	63
Mineral/Vitamin/choline mixture	59
Antioxidant (TBHQ)	0.04
Cholesterol	1.5
Total	1000
Energy density*, kcal/g	4.5
% kcal from Protein	14%
Carbohydrate	44%
Fat	42%
Total	100%
Cholesterol**,%	0.2%

*Values are calculated from ingredient data

**Values are calculated from cholesterol added and from fat source

After overnight fasting, all liver specimens were collected under low-temperature conditions (4 °C) and categorized according to hepatic lobes. To minimize degradation risks, samples were divided into single doses and rapidly frozen with liquid nitrogen, then stored at -80 °C. All specimens were weighed, preprocessed, and subjected to analytical procedures in parallel. Strict protocols were followed to ensure that all samples underwent only a single freeze-thaw cycle throughout the experimental workflow.

### LC-MS-based nontargeted metabolomics profiling

Metabolomics analysis was performed as previously described [21]. Briefly, 80% methanol containing internal standards was added to 10 mg of liver tissues, followed by grinding and homogenization. After vortex mixing, the extract was precipitated, and proteins were removed by centrifugation. The extract was lyophilized and then reconstituted with a water/methanol mixture (4:1, v/v). After dissolving and centrifuging, the supernatant was transferred into a LC-MS system for analysis.

The internal standards used for metabolomics analysis and their final concentrations were as follows: Carnitine C2:0-d3 (0.03ug/mL), Carnitine C10:0-d3 (0.02ug/mL), Carnitine C16:0-d3 (0.025ug/mL), LPC19:0 (0.125ug/mL), FFA C16:0-d3 (0.4ug/mL), FFA C18:0-d3 (0.4ug/mL), CA-d4 (0.3 ug/mL), CDCA-d4 (0.3ug/mL), Phe-d5 (0.5ug/mL), Leu-d3 (0.7ug/mL), Trp-d5 (0.6ug/mL).

Measurement was performed using ultra-performance liquid chromatography (UHPLC) (Shimadzu) coupled with a Triple TOF 5600 plus mass spectrometer (AB SCIEX, Framingham, USA) system. A Waters BEH C8 column (2.1 mm x 50 mm, 1.7 μm) and HSS T3 column (2.1 mm x 50 mm, 1.8 μm) was used for separation in positive and negative modes, respectively. The flow rate was set to 0.4 mL/min and the column temperature was maintained at 60 °C. The elution gradient started at 5% B, held for 0.5 min, then increased linearly to 40% B at 2.0 min, reached 100% B at 8.0 min, and held at this concentration for 2 min. Finally, the column was returned to 5% B within 0.1 min and held for 1.9 min for equilibration. In positive mode, the mobile phases consisted of water (0.1% formic acid, phase A) and acetonitrile (0.1 mM formic acid, phase B). In negative mode, the mobile phases consisted of water (6.5mM ammonium bicarbonate, phase A) and 95% methanol (6.5mM ammonium bicarbonate, phase B).

For mass spectrometry (MS) parameters, the flow rates of sheath gas and curtain gas were set to 55 and 35 psi, respectively. The scan range was set to m/z 100–1250 with a collision energy of 10 V. For dd-MS2 mode, the scan range was set to m/z 50–1250 with collision energy at 35 ± 15 V. In positive ion mode, the ion spray voltage floating was set to 5.5 kV, and the capillary temperature was 550 °C. In negative ion mode, the ion spray voltage floating was − 4.5 kV, and the capillary temperature was 450 °C.

### LC-MS-based nontargeted lipidomics analysis

Lipidomics analysis was conducted according to a previously described method [22]. Lipid extraction was performed using the MeOH/H_2_O/MTBE technique [23]. Briefly, 300 µL of methanol (MeOH) containing internal standards was added to 10 mg of liver tissue, followed by homogenization. Then, 1 mL of methyl tert-butyl ether (MTBE) was added, and the mixture was vortexed for 10 min. Next, 300µL of H_2_O was added, and the mixture was vortexed to form a two-phase system. After centrifugation. 400 µL of the supernatant was lyophilized and stored at -80 °C. Prior to analysis, the lyophilized samples were reconstituted with ACN/IPA/H_2_O (65:30:5, v/v/v/) containing 5 mM ammonium acetate. A 5 µL aliquot of the sample was transferred into the LC-MS system for analysis.

The internal standards used for lipidomics analysis and their final concentrations were as follows: PC 38:0 (1.67ug/mL), PE 34:0 (0.83ug/mL), LPC 19:0 (0.67ug/mL), SM 12:0 (0.83ug/mL), TG 45:0 (1.33ug/mL), Cer 17:0 (0.33ug/mL), FFA 16:0-d3 (0.67ug/mL), FFA 18:0-d3 (0.67ug/mL).

Lipidomics analysis was performed using a Waters ACQUITY UHPLC (Shimadzu) coupled with an AB SCIEX Triple Q-TOF 5600 Plus (Concord, Canada). A Waters BEH C8 column (2.1 mm x 100 mm, 1.7 μm) was used for lipid separation. The mobile phases consisted of 3:2 (v/v) acetonitrile (ACN)/H_2_O (10 mM AcAm, phase A) and 9:1 (v/v) isopropanol (IPA)/ACN (10 mM AcAm, phase B). The flow rate was set to 0.26 mL/min, and the column temperature was maintained at 55 °C. The elution gradient started at 32% B, was held at this concentration for 1.5 min, then increased linearly to 85% B at 15.5 min, reached 97% B at 15.6 min, and was held at this concentration for 2.4 min. Finally, the column was returned to 32% B within 0.1 min and held for 1.9 min for equilibration. The ion spray voltage for MS was set to 5500 V and 4500 V in positive and negative ion modes, respectively. The interface heater temperature was set to 500 °C in positive mode and 550 °C in negative mode. The flow rates for ion source gas 1, ion source gas 2, and curtain gas were set to 50, 50, and 35 psi in positive ion mode and 55, 55, and 35 psi in negative ion mode, respectively. The MS scan range was 300–1250 Da in positive mode and 150–1250 Da in negative mode.

Samples were run in a randomized order to minimize systematic bias. Prior to analysis, several blank injections were performed to ensure baseline stability and clean system conditions. Quality control (QC) samples were analyzed as the first ten injections to condition and stabilize the mass spectrometry instruments. In the batch of real samples, each blank sample and each QC sample were injected after every 10 real samples.

### Data pre-processing

Raw LC-MS data were processed using MS-DIAL (Ver.4.90) software and our lab’s metabolite and lipid database [24]. Compounds were identified based on their accurate mass, chromatographic retention time and MS/MS fragmentation patterns.

For metabolomics analysis, 196 metabolite ion features in positive ionization mode and 138 metabolite ion features in negative ionization mode were identified. And for lipidomics analysis, 385 lipid ion features in positive ionization mode and 187 lipid ion features in negative ionization mode were identified. After removing compounds detected in both ionization modes, and those with low signal-to-noise ratio and high coefficient of variation, 118 polar metabolites and 426 unique lipid species were retained. Multiquant software was used for quantification of raw data. The metabolites and lipids in each sample were normalized based on their corresponding internal standards and the tissue weight.

### Statistical analysis

Data quality was assessed by evaluating the distribution of QC samples on the principal component analysis (PCA) score plot and calculating the relative standard deviation (RSD) of the detected metabolites in QC samples. The data was first performed by median normalization, log transformation and auto scaling. PCA and partial least squares discriminant analysis (PLS-DA) were performed using MetaboAnalyst 6.0 (https://new.metaboanalyst.ca/). In the PLS-DA analysis, cross-validation (k = 5) was applied to optimize the number of latent variables and assess the predictive ability of the PLS-DA model. The Q² value indicated robust generalizability (Q² > 0.5). Additionally, permutation testing (*n* = 1000) confirmed the statistical significance of the model (*p* < 0.05), demonstrating that the observed class separation was not due to chance. These steps ensured both the reliability and validity of our model. Unpaired t-test was applied to determine differentially expressed metabolites (DEMs) between each feeding time and controls within each lobe. The Benjamini-Hochberg method was used to control the false discovery rate (FDR) with a threshold of < 0.05. All the lipid subclasses and specific metabolites in each liver lobe of different groups were visualized on a heat map using the MetaboAnalyst 6.0. DEMs-associated pathway enrichment analysis was also proformed with MetaboAnalyst 6.0. One-way ANOVA was used to identify differences among different interventions. Post-hoc analysis was conducted using the Least Significant Difference (LSD) test to perform pairwise comparisons. Linear mixed models (LMM) were employed to explore explore the impact of different dietary interventions and feeding durations on the metabolic differences among multiple liver lobes. We treated the metabolite measurements from different liver lobes (L1, L2, L3, L4, L5) as five repeated measures and calculated the numbers of differential compounds across liver lobes to evaluate the impact of interventions on the variation among liver lobes. The LMM was structured with different dietary interventions as the fixed effects and included interactions between dietary interventions and liver lobes to assess their impact on the dependent variable (metabolite levels). The random effects component allowed for individual variability by permitting each mouse to have different intercepts and slopes, thereby accounting for individual differences. Furthermore, both different dietary conditions and intervention durations were incorporated into the fixed effects of the LMM to control for confounding effects and provide more accurate estimates. The LMM was implemented using the “lmerTest” package in R, with R version 4.3.2. P-values < 0.05 after FDR correction were considered statistically significant. Scatter plots were generated using GraphPad Prism 8 software (GraphPad Software Inc., La Jolla, CA, USA).

## Results

### Mouse liver metabolome and lipidome coverage and data quality assessment

**Fig. 1:**
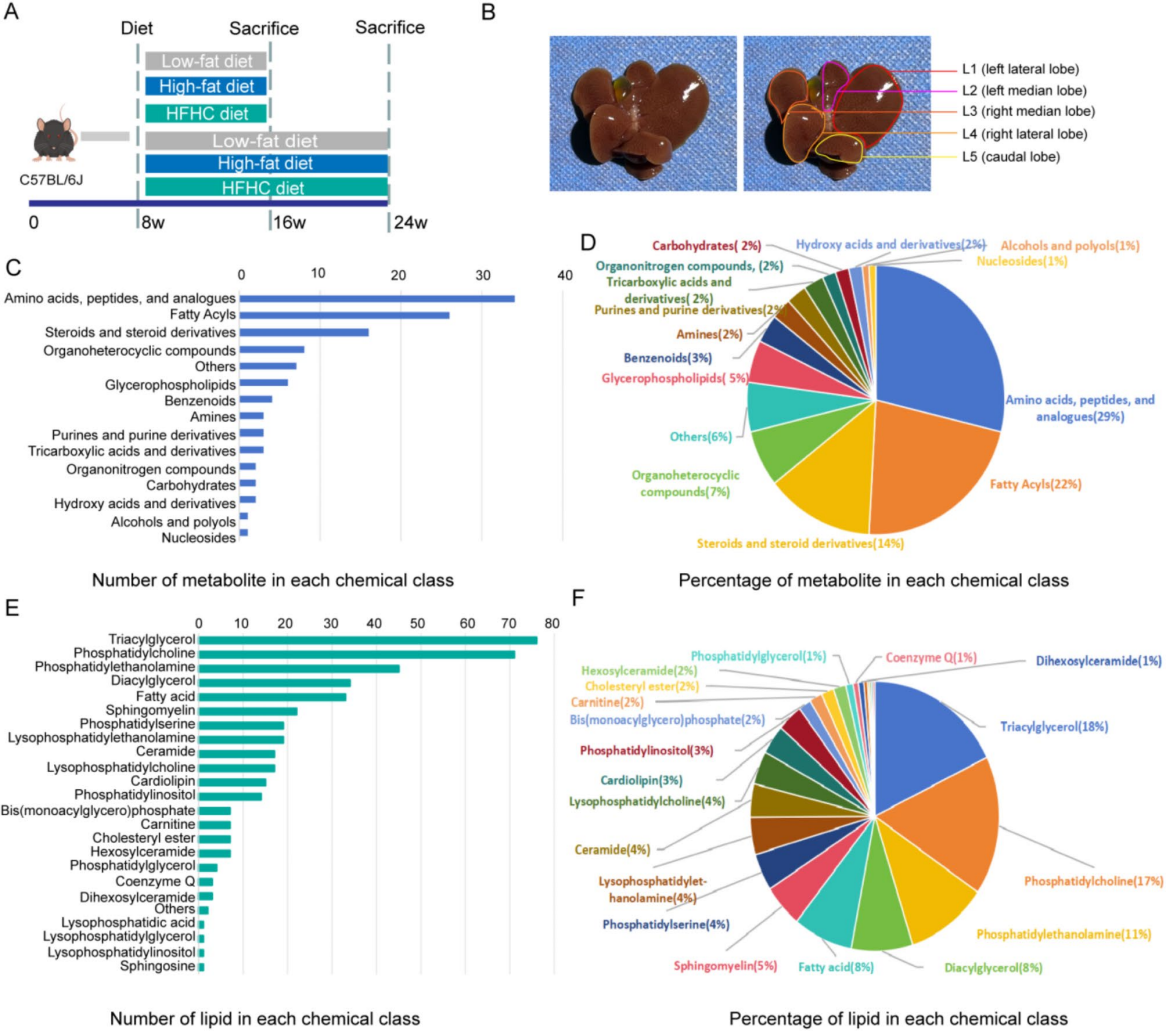
Overview of the mouse liver metabolomics and lipidomics dataset. (**A**) Schematic diagram illustrating the experimental design for mouse liver metabolome dataset. (**B**) Schematic diagram of mouse liver lobes. L1: left lateral lobe (LL); L2: left median lobe (LM); L3: right median lobe (RM); L4: right lateral lobe (RL); L5: caudal lobe (CL). (**C**, **E**) Number of detected metabolites and lipid in each chemical class. (**D**, **F**) Percentage of metabolites and lipids in each chemical class

To depict dynamic metabolic alterations of liver during the onset and progression of MASLD and to investigate the metabolome variations across distinct liver lobes, we performed untargeted metabolomics and lipidomics on five liver lobes of C57BL/6 mice fed HFD or HFHC for different feeding periods (8 weeks or 16 weeks) (Fig. 1A). Control animals were fed a LFD to better understand the specific effects of HFD and HFHC diet [20, 25]. Liver lobes were consistently arranged as shown in Fig. 1B, with each lobe (L1: left lateral lobe (LL), L2: left median lobe (LM), L3: right median lobe (RM), L4: right lateral lobe (RL), L5: caudal lobe (CL)) fully separated for individual analysis. For metabolomics analysis, 196 metabolite ion features in positive ionization mode and 138 metabolite ion features in negative ionization mode were identified using MS-DIAL (ver. 4.93) software and our lab database. For lipidomics analysis, 385 lipid ion features in positive ionization mode and 187 lipid ion features in negative ionization mode were identified using MS-DIAL (ver. 4.93) software [24]. After removing the compounds detected in both ionization modes and those with low signal-to-noise ratio and high coefficient of variation, 118 polar metabolites and 426 unique lipid species were retained (Supplementary Data 1, Supplementary Data 2). According to the Human Metabolome Database (HMDB) classification schemes, we categorized 118 polar metabolites and calculated the number and percentage in each chemical category (Fig. 1C, D). For lipids, we categorized 426 lipids into 25 lipid subclasses based on the Lipidmaps classification scheme and calculated their number and percentage (Fig. 1E, F). These data demonstrated that our study provided sufficient coverage and depth to profile the liver metabolome.

To monitor analytical variability and system stability during the LC-MS analysis, QC sample was generated by combining equal aliquots from all preprocessed experimental samples. The QC sample was injected at regular intervals (every 10 sample injections) throughout the analytical sequence. To visualize dataset variability, two-dimensional PCA score plots were generated for ion features across all ionization modes (positive/negative ionization). The PCA demonstrated tight clustering of QC samples within a 95% confidence ellipse, indicating minimal residual technical variability and robust analytical consistency (Supplementary Fig. 1). This outcome confirmed the absence of significant batch effects or instrumental drift during the acquisition process. The pooled QC samples exhibited RSD of < 30% for over 90% of detected metabolites, confirming strong instrument stability and reliable data in the LC-MS analysis.

To assess variation among all samples, the overall differences and similarities in lipids and metabolites of MASLD mice model were assessed using the supervised multivariate statistical method, PLS-DA. It was observed that the effect of HFHC diet on the metabolic profile of mice was stronger than that of HFD. Furthermore, the lipidome appeared to be more influenced than the metabolome in both models. Compared with 8-week HFD (8HFD) group, those fed HFD for 16 weeks (16HFD) displayed a more pronounced separation from controls in both lipidome and metabolome, suggesting that prolonged HFD feeding significantly exacerbates metabolic disorders. Whereas the mice of 8-week HFHC diet (8HFHC) overlapped with the 16-week HFHC diet (16HFHC) group, indicating that early and intense metabolic disturbances can be triggered by 8 weeks of HFHC feeding (Supplementary Fig. 2A, B). These results suggested that HFD and HFHC diets disrupted metabolic profile of the liver to varying degrees.

### Investigating metabolic profile changes among liver lobes of MASLD mice

**Fig. 2:**
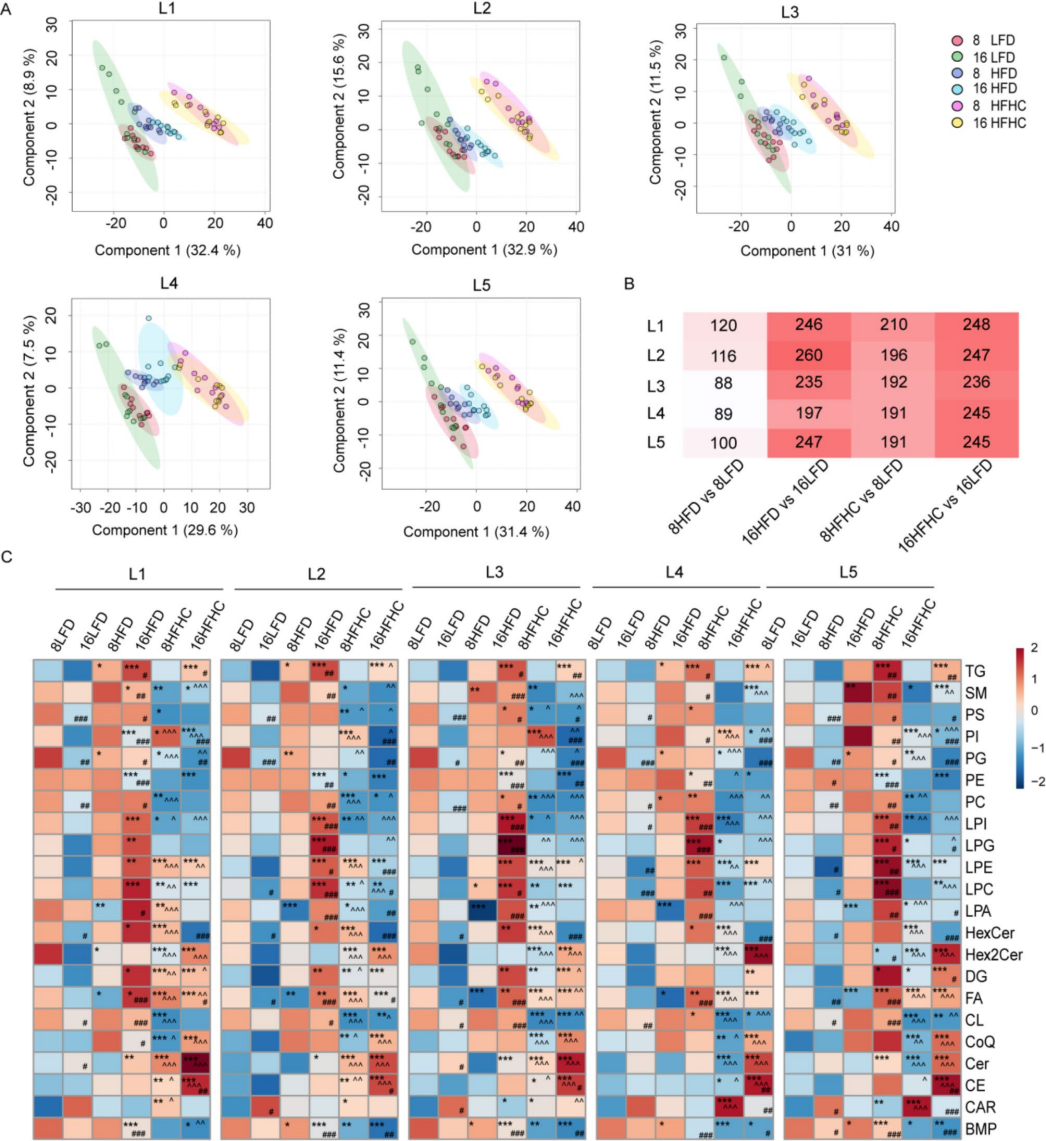
The HFD and HFHC diets trigger time-dependent changes in the hepatic lipidome and metabolome. (**A**) PLS-DA analysis of lipids and metablites in L1, L2, L3, L4, L5 lobes (R2, Q2>0.8). (**B**) Number of DEMs after various feeding periods of HFD and HFHC in 5 liver lobes. (**C**) Heat map of the differentially expressed metabolites (detected by lipidome) among 5 liver lobes in mice fed HFD and HFHC for 8 weeks or 16 weeks. One-way ANOVA was used to identify differences among different interventions. Post-hoc analysis was conducted using the LSD test to perform sufficient comparisons. *indicates comparisons between dietary interventions and the corresponding time-point LFD (8HFHC/8HFD vs. 8LFD, 16HFHC/16HFD vs. 16LFD). # indicates comparisons between different time points under the same dietary intervention (16LFD vs. 8LFD, 16LFD vs. 8LFD, 16HFHC vs. 8HFHC). ^indicates comparisons between HFHC and HFD at same feeding time (8HFHC vs. 8HFD, 16HFHC vs. 16HFD). */#/^ *P* < 0.05, **/## /^^*P* < 0.01, ***/###/^^^ *P* < 0.001

To gain insights into metabolic profile changes across liver lobes with different feeding methods and durations, we applied PLS-DA to identify altered lipids and metabolites across liver lobes at varing feeding methods and durations. Consistent with the whole liver analysis, the 16HFD group displayed more pronounced separation from controls than the 8HFD group, and this pattern was consistent across all liver lobes. Unlike the HFD, which primarily induces simple steatosis, the HFHC diet promotes progression to liver inflammation, hepatocyte damage and fibrosis with prolonged feeding [26, 27]. Across all liver lobes, the PLS-DA model clearly separated the 8HFHC group from the LFD group, while showing overlap with the 16HFHC group (Fig. 2A). Furthermore, the number of DEMs in each liver lobe indicated that metabolic differences induced by HFD and HFHC intensified over time. Notably, in the 8HFD and 8HFHC groups, L1 had significantly more DEMs than the other liver lobes, suggesting that the metabolic profiles of the liver lobes may be inconsistent early stage of MASLD progression but tended to stabilize with extended feeding (Fig. 2B).

We then analyzed the lipidome and metabolome components separately. Lipid subclasses were quantified to evaluate changes in lipids under various dietary conditions. The heatmap showed distinct metabolic patterns of lipids in HFHC and HFD. Compared with the HFD group, the HFHC group displayed differences in various lipids, including bis(monoacylglycero)phosphate (BMP), cholesteryl ester (CE), ceramide (Cer), coenzyme Q (CoQ), cardiolipin (CL), diacylglycerol (DG), fatty acid (FA), dihexosylceramide (Hex2cer), hexosylceramide (HexCer), lysophosphatidic acid (LPA), lysophosphatidylcholine (LPC), lysophosphatidylethanolamine (LPE), lysophosphatidylinositol (LPI), phosphatidylglycerol (PG), phosphatidylinositol (PI), and sphingomyelin (SM). Specifically, multiple lipid subclasses showed progressive increases over HFD feeding time, including FA, LPA, LPC, LPE, LPI, lysophosphatidylglycerol (LPG), DG, and triacylglycerol (TG), which align with the characteristics of MASLD. Conversely, phosphatidylethanolamine (PE) and BMP levels decreased significantly over time, while phosphatidylserine (PS) and phosphatidylcholine (PC) levels increased at certain time point (Fig. 2C). These results indicated that HFD livers exhibited increases in glycerolipids and lysolecithins, alongside decreases in phosphoglycerides and BMP over time. Significant alterations in lipids were also observed during HFHC exposure. In detail, the levels of CoQ, Cer, CE, DG, FA, LPE, Hex2Cer, and TG significantly increased with MASLD severity, while BMP, HexCer, PE, PG, and PI decreased with feeding duration. Additionally, the levels of CL, LPA, LPC, LPI, LPG, PC, PS, and SM were reduced in the early stages of HFHC feeding (Fig. 2C). These results suggested that HFHC-fed livers, in addition to exhibiting steatosis, displayed reduced levels of BMP, HexCer, CL, and phosphoglycerides, along with elevated levels of CoQ, Cer, LPE, and Hex2Cer, aligning with oxidative stress and mitochondrial dysfunction characteristic of MASH [28–30].

**Fig. 3:**
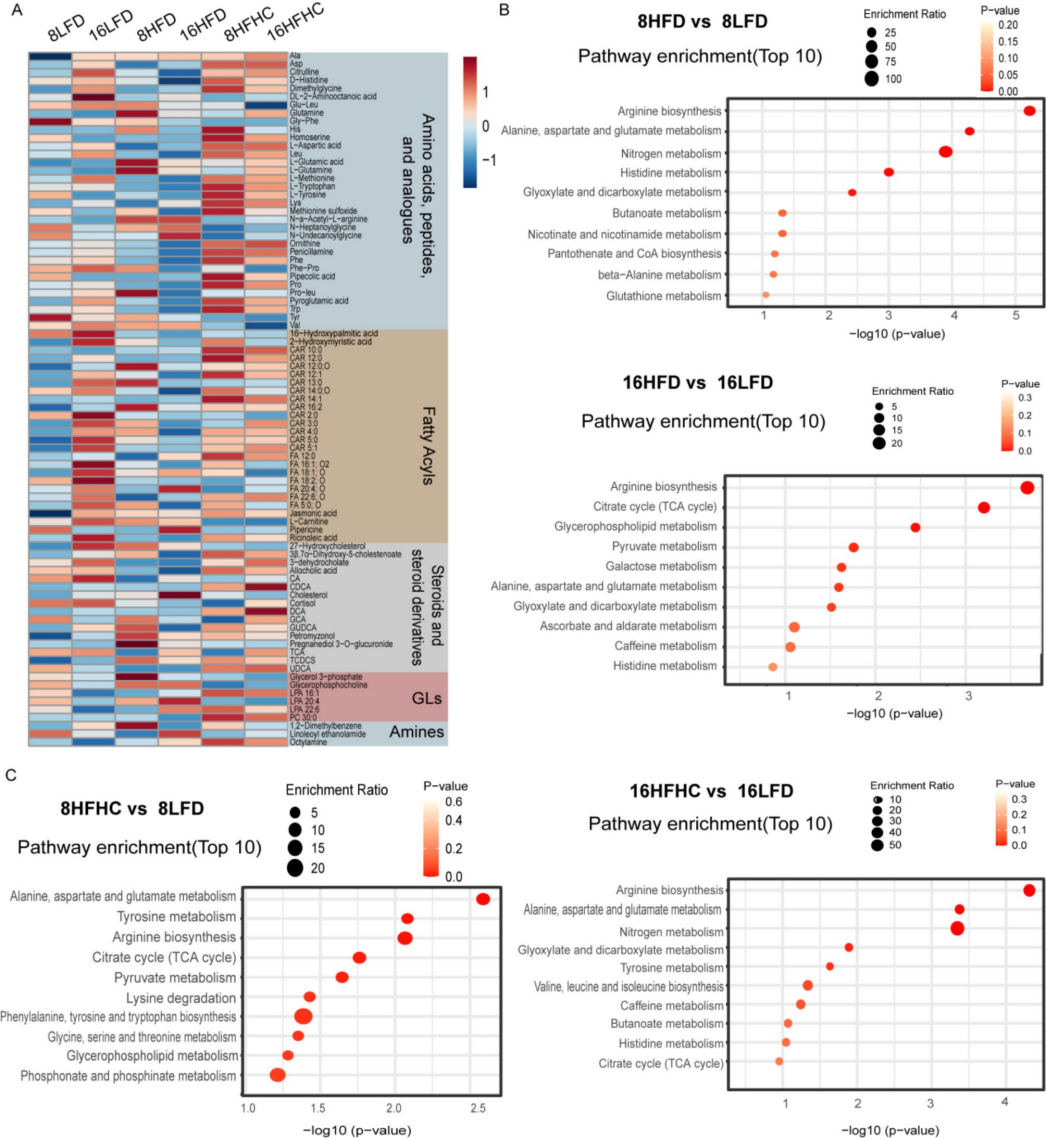
Changes in the hepatic metabolome in mice fed HFHC diet at 8 weeks and 16 weeks. (**A**) Heat map of the differentially expressed metabolites (detected by metabolome) of L1 lobe only. (**B**) Top-10 metabolic pathways (metabolome data) that were associated with HFD at different times (8HFD vs. 8LFD; 16HFD vs. 16LFD) in L1 lobe. (**C**) Top-10 metabolic pathways (metabolome data) that were associated with HFHC at different times (8HFHC vs. 8LFD; 16HFHC vs. 16LFD) in L1 lobe. The enrichment ratio represents the number of DEMs within the specified pathway. A larger circle indicates a higher degree of enrichment. Pathway enrichment analysis was performed utilizing the Pathway Analysis module from online MetaboAnalyst 6.0 (https://new.metaboanalyst.ca/)

For metabolome, global alterations in metabolites showed similar metabolic patterns across all liver lobes under dietary stimulation (Supplementary Fig. 3, Supplementary Fig. 4). We then performed differential metabolite analysis and pathway analysis with L1 as the main target. The heatmap showed distinct metabolic patterns of metabolites in HFHC and HFD. Notably, the compounds in the amino acid class seem to have more pronounced metabolic changes at 8HFHC than 16HFHC (Fig. 3A). Pathway enrichment analysis revealed that early HFD feeding primarily affects amino acid metabolic pathways. As the duration of HFD feeding extends to 16 weeks, arginine biosynthesis, citrate cycle, and glycerophospholipid metabolism emerged as the most representative pathways (Fig. 3B). These identified pathways suggested that HFD disrupted cellular amino acid metabolism, energy metabolism and lipid metabolism, thereby increasing energy supply and storage in the liver. For the mice in the early stages of HFHC feeding (8HFHC group), pathway enrichment analysis identified significant alterations in multiple amino acid pathways, including alanine, aspartate and glutamate metabolism, tyrosine metabolism, and arginine biosynthesis. As the duration of HFHC feeding extended to 16 weeks, alanine, aspartate and glutamate metabolism, nitrogen metabolism, glyoxylate and dicarboxylate metabolism showed significant alterations (Fig. 3C). The results indicated that amino acid metabolic pathways were significantly impacted in the livers of MASLD mice, particularly in the early stages.

### Remodeling of amino acid metabolism during MASLD progression

The results above demonstrated that amino acid metabolism was disrupted in HFHC-fed mice, prompting us to further investigate the important role of amino acid metabolism in the progression of MASLD into MASH induced by HFHC diet.

**Fig. 4:**
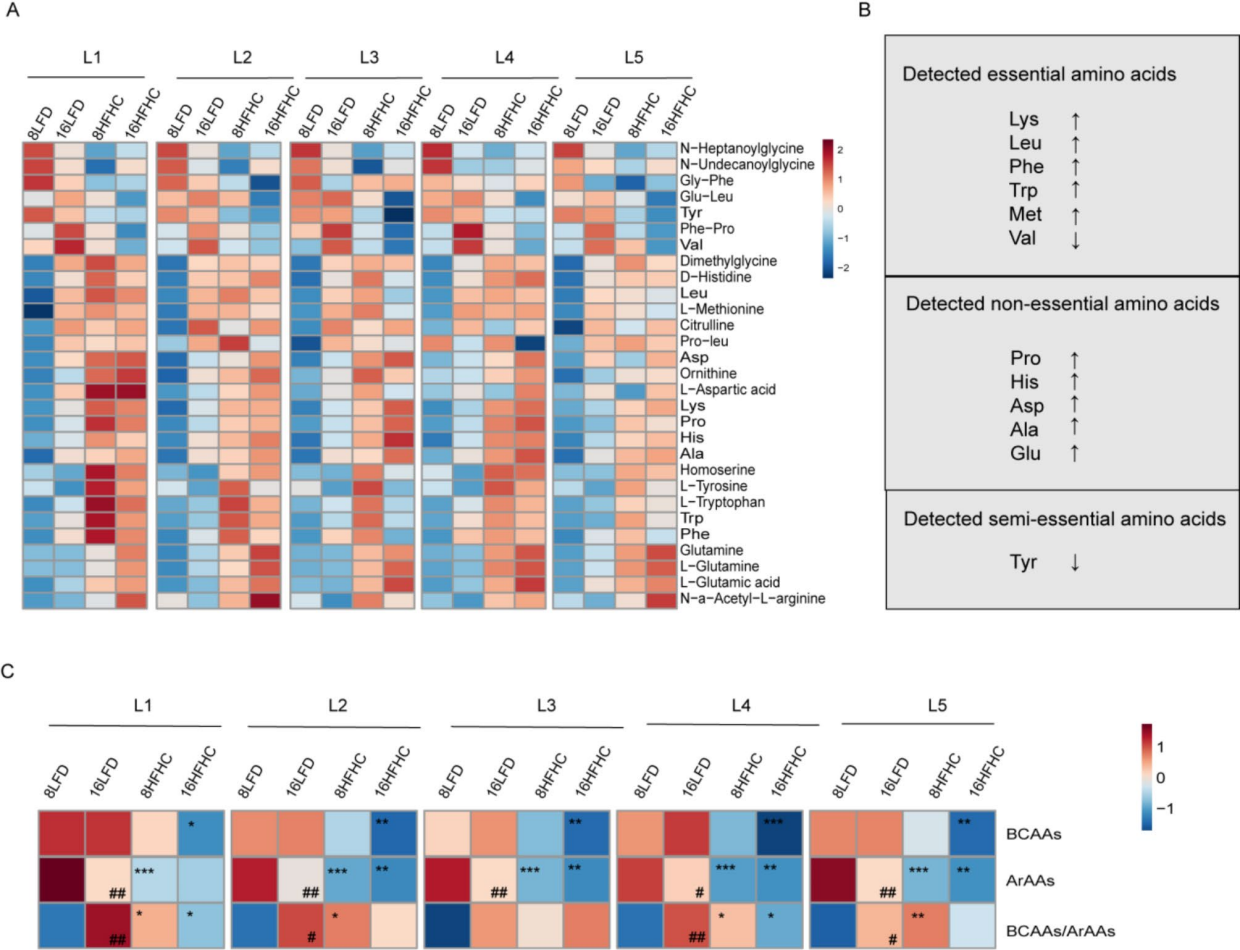
Changes of amino acid among five liver lobes in MASLD progression stage. (**A**) Heat map of all amino acid levels after different periods of HFHC diet in 5 liver lobes. (**B**) The classification of detected amino acids and statistics on the trend of changes in various amino acids during the progression of MASLD. (**C**) Heat map of BCAAs, ArAAs levels and BCAAs/ArAAs ratios of HFHC mice at 8weeks and 16weeks feeding. *indicates comparisons between dietary interventions and the corresponding time-point LFD (8HFHC vs. 8LFD, 16HFHC vs. 16LFD). # indicates comparisons between different time points under the same dietary intervention (16LFD vs. 8LFD, 16HFHC vs. 8HFHC). */# *P* < 0.05, **/## *P* < 0.01, ***/### *P* < 0.001

Overall, the levels of most detected amino acids were elevated across the five liver lobes of HFHC mice, except for valine and tyrosine (Fig. 4A, B). Branched-chain amino acids (BCAAs) including leucine, isoleucine, and valine, along with aromatic amino acids (ArAAs), such as phenylalanine and tyrosine, are among the most studied amino acids, as their plasma and liver levels reflect liver pathophysiology [31, 32]. In HFHC-fed mice, valine and tyrosine levels decreased, while leucine, phenylalanine, and tryptophan levels increased across all five liver lobes. The BCAAs/ArAAs ratio also significantly increased in 8HFHC (Fig. 4C). Additionally, amino acids involved in the urea cycle showed altered levels [33]. Citrulline and ornithine were increased, while hepatic acetyl-L-arginine, a compound elevated in hyperargininemia, was also elevated in HFHC-fed mice [34]. These changes suggested altered urea synthesis, increased ornithine cycle activity, and enhanced substrate availability for the citrate cycle. Excessive consumption of fat-rich diets leads to increased ammonia production from amino acid metabolism, which stimulates the synthesis of enzymes in the ornithine cycle, thereby enhancing ornithine metabolism. Notably, several amino acids, such as lysine, proline, histidine, and aspartic acid, were elevated in the L1 lobe of 8HFHC group compared to the other lobes (Fig. 4A). This metabolic difference between liver lobes was not significant in 16-week HFHC mice, suggesting that the L1 was more susceptible to amino acid metabolism disruptions in the early stages of HFHC feeding.

### High-nutrient diets exacerbate metabolic variation among liver lobes

To elucidate the impact of two distinct dietary regimens over varying durations on metabolic differences between liver lobes, we employed a LMM, treating metabolite measurements from different liver lobes (L1, L2, L3, L4, L5) as five repeated measures. This approach allowed us to account for both fixed effects (dietary regimens) and random effects (individual differences among mice). We evaluated the impact of interventions on the variation among liver lobes by calculating the numbers of differential compounds across liver lobes. As shown in Tables 3 and 8HFD, 8HFHC, 16HFD and 16HFHC groups caused differences in the levels of 249, 308, 294, and 275 compounds among liver lobes compared to LFD group, respectively. Compared to the corresponding HFD at the same time point, 8HFHC and 16HFHC caused differences in the levels of 356 and 244 compounds among liver lobes. Moreover, for the same diet, the numbers of differential compounds between liver lobes in the 16-week feeding group compared to the 8-week feeding group were as follows: 92 for LFD, 163 for HFD, and 50 for HFHC (Supplementary Data 3). Approximately 90% of above differential compounds were lipids rather than other metabolites. We further analyzed the number of differential lipid subclasses among lobes and found that, compared to the 8LFD group, 8HFHC affected 16 lipid subclasses, a significantly higher number than others (Table 3, Supplementary Data 4).

**Table 3:** Assessing the impact of interventions on the variation among liver lobes using linear mixed model

Comparison Groups	Number of variable metabolites and lipids between liver lobes	Number of variable lipids between liver lobes	Number of variable metabolites between liver lobes	Number of variable lipid subclasses between liver lobes
8LFD vs. 8HFD	249	221	28	3
8LFD vs. 8HFHC	308	295	13	16
8HFD vs. 8HFHC	356	312	44	14
16LFD vs. 16HFD	294	265	29	9
16LFD vs. 16HFHC	275	263	12	11
16HFD vs. 16HFHC	244	232	12	10
8LFD vs. 16LFD	92	80	12	4
8HFD vs. 16HFD	163	144	19	5
8HFHC vs. 16HFHC	50	48	2	3

We evaluated the impact of feedings on the variation among liver lobes by calculating the numbers of differential compounds across liver lobes. The numbers indicated the quantity of differential compounds across liver lobes between different groups. Compounds were considered statistically significant if the p-value (FDR correction) < 0.05. Detailed information for each compound is provided in the Supplementary Data 3, 4

Our analysis revealed that high-nutrient diets (HFD and HFHC) significantly induced metabolic heterogeneity among liver lobes, particularly in lipids. The observed increase in metabolic variation among liver lobes may reflect differential sensitivities to nutrient overload within each lobe. Additionally, the 8-week HFHC-fed mice had the most significant impact on lipid differences among liver lobes.

### Exploring lipid alterations in different lobes of MASLD mice liver

**Fig. 5:**
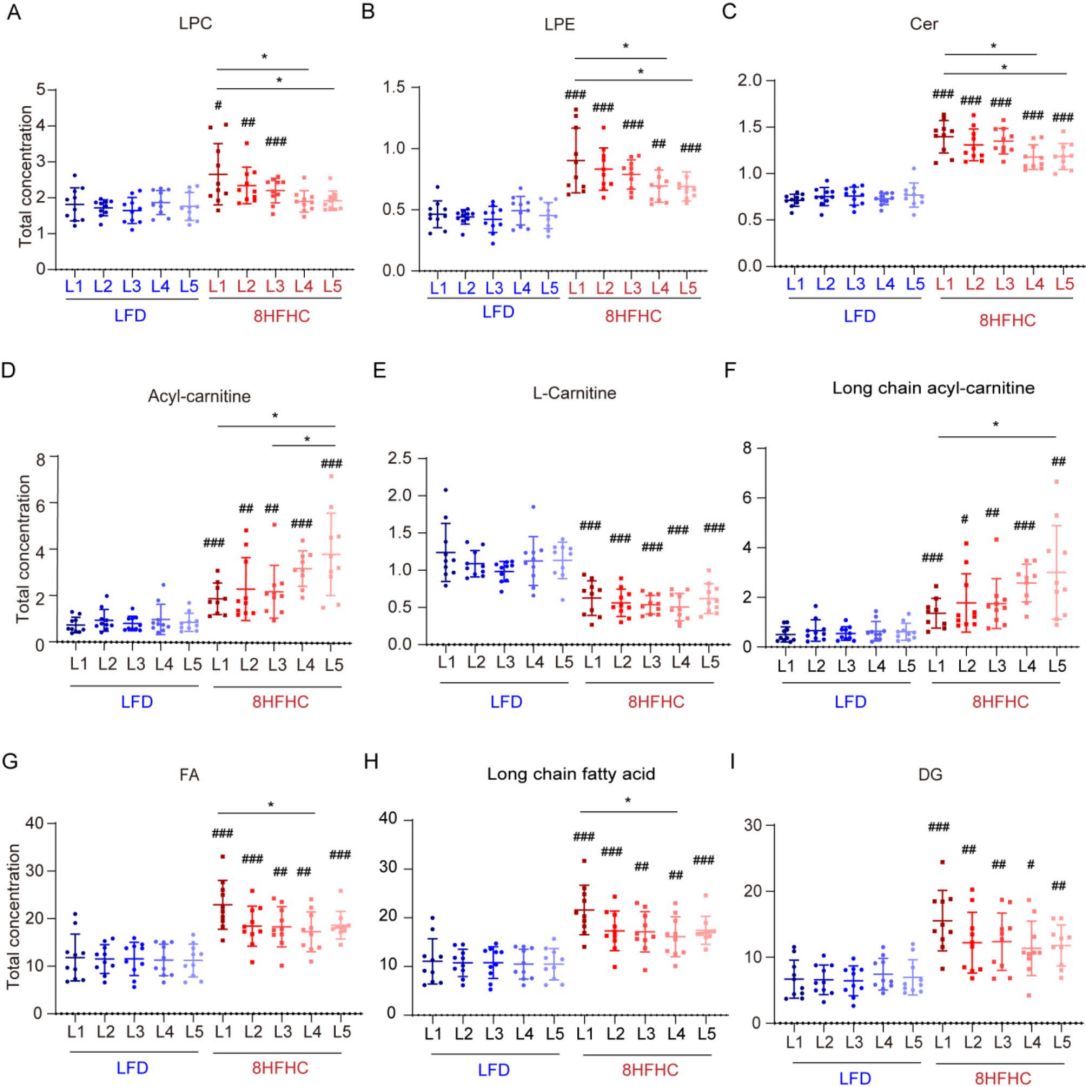
Changes of lipids among five liver lobes in MASLD progression stage. Changes in LPC (**A**), LPE (**B**), Cer (**C**), acyl-carnitine (**D**), L-carnitine (**E**), long chain acyl-carnitine (**F**), FA (**G**), long chain FA (**H**), and DG (**I**) contents in different liver lobes of LFD and 8 weeks HFHC diet mice. *indicates comparisons among liver lobe within the same diet. # indicates comparisons between two groups within the same liver lobe. */# *P* < 0.05, **/## *P* < 0.01, ***/### *P* < 0.001

To determine whether specific lipids exhibit distinct behaviors across liver lobes, we quantified lipid subclasses and found that HFHC feeding induced differential levels of LPC, LPE, and Cer across lobes. Specifically, LPC, LPE, and Cer levels were significantly lower in lobes L4 and L5 compared to L1 (Fig. 5A-C). As lysophospholipids (LPLs) from different substrates, LPC is a biologically active pro-inflammatory molecule produced through pathological activity [35, 36], while elevated LPE levels are associated with several metabolic diseases [37]. Cer are lipotoxic and pro-inflammatory, promoting reactive oxygen species (ROS) generation through mitochondrial oxidative respiration [38, 39]. Although the levels of these three lipids were elevated in response to HFHC diet, they remained significantly lower in the lobes L4 and L5 than in lobe L1, suggesting a milder inflammatory response and oxidative stress in L4 and L5.

In contrast, acyl-carnitine, generated by esterification of L-carnitine with fatty acids, was significantly higher in lobes L4 and L5 compared to L1. Meanwhile, the levels of free L-carnitine, which is physiologically active, decreased with HFHC feeding and showed no significant difference among liver lobes (Fig. 5D, E). Long-chain fatty acids must bind to carnitine to form acylcarnitine, enabling mitochondrial membrane transport for β-oxidation [40, 41]. We further classified acyl-carnitines according to the carbon chain length of their fatty acids. Analysis of varying acyl-carnitines revealed that long-chain acyl-carnitines, rather than short-, medium-, or very-long-chain types, differed significantly across liver lobes (Fig. 5F, Supplementary Fig. 5A-C). It implied that more long-chain fatty acids bound to free carnitine entered the mitochondria for β-oxidation in the lobe L5. We then classified the fatty acids based on carbon chain lengths. As expected, long-chain fatty acids were significantly lower in lobes L4 and L5 compared to L1 (Fig. 5G, H, Supplementary Fig. 5D, E). Additionally, the HFHC diet-induced increase in DG was most pronounced in lobe L1, although it was not statistically significant(Fig. 5I). Notably, by the 16w HFHC diet, these lipid differences across lobes were substantially reduced compared to the 8HFHC group (Supplementary Fig. 5F).

In conclusion, comparison of lipid profiles between HFHC and control groups revealed that L1 was more susceptible to early HFHC-induced lipid accumulation, inflammation, oxidative stress, and mitochondrial β-oxidation than the other lobes during MASLD progression, with lipid distributions across lobes converging over time.

## Discussion

MASLD is an escalating metabolic disorder that poses a significant global health challenge [2]. Investigating the metabolomic profile of MASLD is essential for understanding disease progression and identifying potential therapeutic targets. In this study, we generated a comprehensive metabolome atlas of MASLD in mice fed either a HFD or HFHC diet, analyzing metabolic profiles across different liver lobes using metabolomics and lipidomics. Our results confirmed prior findings that lipid and amino acid metabolism undergoes extensive remodeling in the mouse liver during MASLD progression due to overnutrition. Notably, this study is the first to report lobe-specific metabolic responses, with the L1 liver lobe showing heightened sensitivity to lipid and amino acid alterations during disease progression. These insights into hepatic metabolic characteristics across liver lobes provide a valuable resource for understanding the full spectrum of MASLD.

Simple steatosis and steatohepatitis represent two distinct pathological states within the spectrum of MASLD. The pathological changes induced by HFHC are dynamic and continuous. In the early stage of feeding (4–8 weeks), simple steatosis is mainly observed in the liver. As the feeding duration extends to 16 weeks, liver inflammation and injury are significantly exacerbated, manifesting as MASH. Therefore, the high cholesterol content of HFHC accelerates the development of liver inflammation and fibrosis, making it an ideal model for studying the progression from simple steatosis to MASH and capturing the full pathological spectrum of MASLD [26, 42]. In contrast, the HFD primarily induces significant hepatic lipid accumulation without the marked inflammatory and fibrotic changes. Thus, the HFD is more suitable for studying the mechanisms underlying isolated liver steatosis and its metabolic implications [27, 43]. The structure of the mouse liver consists of five separate lobes including the left lateral lobe, left median lobe, right median lobe, right lateral lobe and caudal lobe [44]. Previous studies on the effect of diet on the liver metabolome have been restricted to a single identified liver lobe or whole liver tissue, primarily due to consideration of experimental data reproducibility. There have been no studies on metabolite differences between different liver lobes in MASLD model mice. In our study, we divided the mouse liver into five hepatic lobes according to their structural characteristics, and attempted to identify differences in the distribution of specific metabolites within different liver lobes under high-fat conditions.

In our study, we observed significant differences in metabolite and lipid profiles between the HFD and HFHC mouse models. Based on the PLS-DA, lipids were more affected by dietary overload than metabolites, consistent with the accumulation of hepatic fat typical in MASLD [45, 46]. In HFD-fed mice, prolonged feeding led to worsened hepatic lipid accumulation, while HFHC-fed mice showed significant metabolic changes even during the early stages of feeding, indicating that high cholesterol of the diet may accelerate metabolic disorders. The findings align with prior research indicating that earlier and more pronounced metabolic alterations due to the HFHC diet may directly contribute to the pathology associated with MASLD [26]. These findings underscore the necessity of cholesterol-centric interventions during the initial stages of MASLD development.

The pattern of lipid metabolism is altered in MASLD disease. PC, a major phospholipid in mammals, constitutes up to 30% of hepatocyte lipids, largely originating from PE via the Cytidine Diphosphate (CDP)-ethanolamine pathway or from PS through decarboxylation [47]. Decreased levels of these glycerophospholipids, which are essential for cell membrane integrity, increase membrane permeability to pro-inflammatory molecules [48]. While all three glycerophospholipids were reduced in the HFHC model, PC and PS increased in HFD-fed mice, suggesting that glycerophospholipid metabolic pathways differ between simple steatosis and MASH. We also observed a consistent increase in LPE, accompanied by a decrease in PE in HFD-fed and HFHC-fed mice, suggesting disrupted phospholipid turnover. In HFHC-fed mice, in addition to steatosis, ceramide and CoQ levels increased, while CL decreased. Accumulating evidence positions ceramide, CoQ, and CL as critical modulators of mitochondrial homeostasis, with their dysregulation serving as harbingers of progressive mitochondrial dysfunction in MASLD [30, 49, 50]. In our study, we observed that changes in ceramides, CoQ, and CL levels were not transient but rather persistent and stable over time, indicating that they are not short-term metabolic fluctuations but likely reflect mitochondrial oxidative stress and dysfunction during MASLD progression. Interestingly, BMP decreased with feeding time in both steatosis and MASH models. BMP, which promotes acidic sphingomyelinase activity in late endosomes and lysosomes [51, 52], is often elevated in various genetic lysosomal storage disorders (LSD) [53]. The decrease in BMP observed in our study may be linked to functional defects of cell organelles such as endosome and lysosomes systems. However, previous studies have showed that BMP content in liver increased 2-fold and 6-fold in mice fed the atherogenic diet and the obesity-inducing diet, respectively, while no significant changes were observed in mice fed Western diet [54]. These findings are inconsistent with our results, suggesting that further studies are warranted to explore BMP regulation under dietary lipid overload and determine whether BMP modulation could mitigate MASLD progression. Additionally, further research should elucidate the molecular pathways involved in BMP synthesis, degradation, and release.

**Fig. 6:**
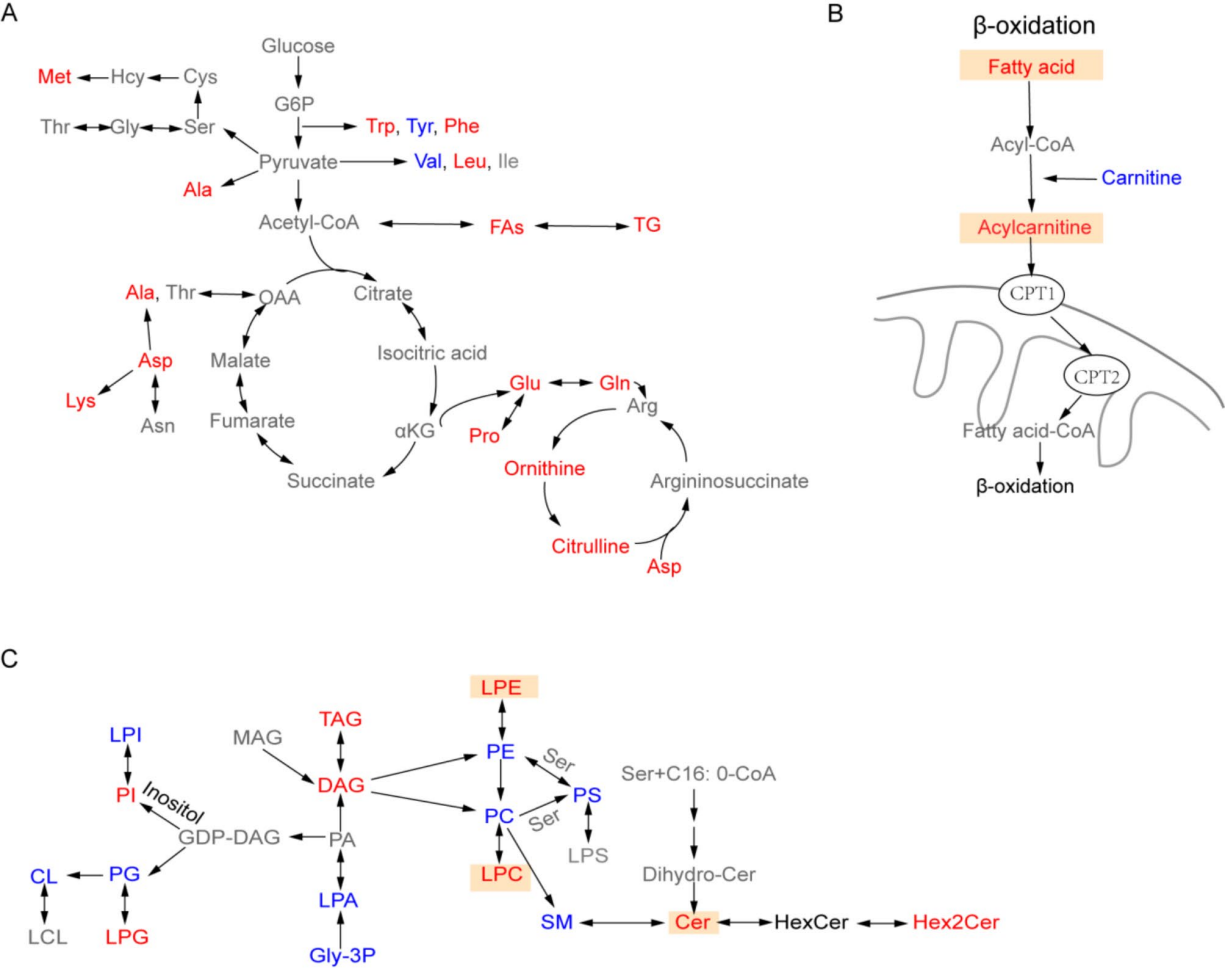
Changes in metabolic processes during the progression of MASLD. (**A**) Changes in amino acids of mice fed HFHC diet. (**B**) Mitochondrial β-oxidation process. (**C**) Changes in lipid metabolic pathways of HFHC mice. Red text represents upregulated metabolites, and green text represents downregulated metabolites when the HFHC group is compared with the LFD group, and gray text represents undetected metabolites. Yellow tag represents the metabolites with differential content in different liver lobes of mice in the 8HFHC group

Our findings highlight the significant disruptions in cellular amino acid and energy metabolism induced by HFD and HFHC diets. These disruptions are integral to the liver’s adaptive response to dietary overload, characterized by increased energy storage and production. The mice fed HFHC diet for 8 weeks exhibited greater enrichment of amino acids, suggesting that changes in amino acid metabolism were more pronounced in MASLD progression, particularly in the early stages (Fig. 6A). Several studies have identified increased levels of BCAAs and ArAAs during the transition from simple steatosis to MASH [55]. Additionally, levels of specific amino acids, such as phenylalanine and its metabolite tyrosine, often decrease in liver disease patients [56, 57]. In this study, valine and tyrosine levels decreased, and the ratio of BCAAs to ArAAs increased. However, whether these changes directly promote MASLD progression or are secondary to liver insulin resistance remains a topic of debate. The alterations in BCAAs and ArAAs can both directly promote MASLD progression through mechanisms involving lipid accumulation and inflammation, and they can also be secondary consequences of liver insulin resistance [58–60]. Future research should focus on elucidating these relationships through a combination of gene editing and clinical intervention trials. Understanding the precise mechanisms underlying these relationships is crucial for identifying potential therapeutic targets that can break the cycle of metabolic dysfunction and disease progression. Notably, during the progression from steatosis to MASH—particularly HFHC diet feeding for eight weeks —these amino acid dysregulations were more pronounced in the L1 liver lobe, suggesting that hepatic lobe L1 in mice may be more sensitive to disturbances in amino acid metabolism.

LMM analysis revealed that high-nutrient diets significantly induced metabolic heterogeneity among liver lobes, particularly in lipids. Additionally, the 8-week HFHC-fed mice had the most significant impact on lipid differences across liver lobes, prompting us to further investigate metabolic differences in lipids during the progression of MASLD. Elevated levels of LPC, LPE, and ceramides in response to HFHC diet stimulation exhibited variability among hepatic lobes, suggesting that L1 may exhibit more severe inflammation and oxidative stress compared to other lobes. Furthermore, fatty acid levels, particularly long-chain fatty acids, were significantly higher in L1 than in L4/L5. It is well established that the liver is the most active tissue for fatty acid oxidation, and its most important form of oxidation is β-oxidation [41]. In this process, fatty acids are activated to acyl-CoA by acyl-CoA synthetase before oxidation. Medium- and short-chain fatty acids can enter mitochondria directly, while long-chain acyl-CoA requires conversion to acyl-carnitine by reacting with carnitine to facilitate transport into the mitochondria. In our study, long-chain acyl-carnitines were significantly lower in L1 than in L5, suggesting that the L5 lobe may be more efficient in facilitating fatty acid breakdown via esterification with carnitine (Fig. 6B). Regarding lipid accumulation, we observed a trend of elevated DG levels in L1 relative to other lobes, suggesting that lipid accumulation might vary by hepatic lobe in the early stages of MASLD progression. Shortening HFHC feeding duration may uncover more differences. Interestingly, these differences diminished when HFHC feeding was extended to 16 weeks. In comparison, no such differences were observed between the liver lobes of HFD-fed mice with simple steatosis. This suggests a consistent response to lipid deposition across liver lobes in simple steatosis. Overall, our findings indicate that, among the five lobes, the left lateral lobe exhibited the greatest metabolic disturbances in metabolites associated with inflammation and mitochondrial β-oxidation during the progression from steatosis to MASH, particularly in the early stage of MASLD (Fig. 6C).

This lobe-specific sensitivity highlights the complexity of hepatic metabolic responses to dietary interventions. The liver is a highly vascularized organ with complex zonation, and its lobes can exhibit regional heterogeneity in metabolism, detoxification, and response to injury [7, 8]. The differential blood supply patterns (portal vs. arterial dominance) and oxygen gradients across lobes create distinct microenvironments that influence lipid metabolism, oxidative stress responses, and inflammatory signaling. As the primary recipient of portal blood, left lateral lobe hepatocytes experience earlier and more intense exposure to diet-derived lipids and gut-derived signals. This increased blood flow results in higher exposure to nutrients, toxins, and metabolic intermediates, making it more susceptible to metabolic disturbances. Additionally, the uneven distribution of metabolic enzymes across liver lobes can lead to regional differences in metabolic activity. For example, enzymes such as fatty acid synthase, acetyl-CoA carboxylase [15] and carnitine palmitoyltransferase I [9], which play key roles in lipid metabolism, may be more highly expressed in certain liver regions. These differences suggest that some regions of the liver may be more susceptible to lipid accumulation or inflammatory processes due to local variations in gene expression. The concept of metabolic heterogeneity across liver regions has important implications for human studies. In humans, the liver is divided into functionally distinct zones based on its blood supply and metabolic activities. Similar to our observations in mice, certain regions of the human liver may exhibit higher metabolic activity and thus be more susceptible to metabolic disturbances [61]. Targeted therapies that address specific metabolic pathways in vulnerable liver regions may be more effective in treating MASLD.

While our study provides valuable insights, these findings should be interpreted with caution due to several limitations. Firstly, anatomical and physiological differences between mice and humans mean that direct translation of our findings to clinical settings requires careful consideration. Further studies are needed to validate these findings in humans, possibly through targeted biopsies or advanced imaging techniques that can assess regional metabolic activity. Secondly, our study primarily relied on observational data and did not include functional experiments to validate the metabolic changes observed across different liver lobes. Future work should include functional studies to elucidate the underlying mechanisms and pathological processes occurring in different liver lobes. Thirdly, the use of a single metabolomics approach to study liver lobe differences may not fully capture the complexity of the hepatic response to dietary overload. Metabolic changes are often interconnected with alterations in gene expression, protein levels, and cellular signaling pathways. Therefore, future research should integrate multiple omics datasets to provide a more comprehensive understanding of liver lobe-specific responses.

## Conclusion

In summary, we have created a detailed metabolome atlas of liver lobes, revealing dynamic metabolic shifts across different liver lobes during the progression from steatosis to MASH using lipidomic and metabolomic techniques. As the disease progresses from steatosis to MASH, we observed a shift in metabolic patterns involving lipids (phospholipids, lysophospholipids, ceramides, cardiolipin) and amino acids (BCAAs, ArAAs). The metabolic heterogeneity across different regions of the liver suggests that the pathogenesis of MASLD may involve region-specific metabolic functions of the liver. Our findings enhance our understanding of liver physiology and pathology by providing a comprehensive characterization of dynamic metabolic changes, and they offer valuable insights for the selection of liver lobes in experimental studies. However, further studies are required to validate these findings in humans and clarify the physiological significance of hepatic metabolic zonation.

## Electronic supplementary material

Below is the link to the electronic supplementary material.

Supplementary Material 1

Supplementary Material 2

Supplementary Material 3

Supplementary Material 4

Supplementary Material 5

## Data Availability

Data is provided within the manuscript or supplementary information files.

## Abbreviations

ArAAs: Aromatic amino acids
Ala: Alanine
Asp: Aspartic acid
ACC: Acetyl-CoA carboxylase
ACN: Acetonitrile
BCAAs: Branched-chain amino acids
BMP: Bis(monoacylglycero)phosphate
CDP: Cytidine Diphosphate
CL: Caudal lobe
CAR: Carnitine
CE: Cholesteryl ester
CL: Cardiolipin
Cer: Ceramide
CoQ: Coenzyme Q
DG: Diacylglycerol
DEMs: Differentially expressed metabolites
FA: Fatty acid
FASN: Fatty acid synthase
FDR: False discovery rate
Glu: Glutamic acid
HFD: High-fat diet
HFHC: High-fat/high-cholesterol
HMDB: Human Metabolome Database
Hex2Cer: Dihexosylceramide
HexCer: Hexosylceramide
His: Histidine
IPA: Isopropanol
LFD: Low-fat diet
LC-MS: Liquid chromatography–mass spectrometry
LL: Left lateral lobe
LM: Left median lobe
LMM: Linear mixed model
LPA: Lysophosphatidic acid
LPC: Lysophosphatidylcholine
LPE: Lysophosphatidylethanolamine
LPG: Lysophosphatidylglycerol
LPI: lysophosphatidylinositol
Lys: Lysine
Leu: Leucine
LSD: Lysosomal storage disorders
LPLs: Lysophospholipids
MASLD: Metabolic dysfunction-associated steatotic liver disease
MASH: Metabolic dysfunction-associated steatohepatitis
Met: Methionine
MS: Mass spectrometry
MeOH: Methanol
MTBE: Methyl tert-butyl ether
NAFLD: Non-alcoholic fatty liver disease
PCA: Principal component analysis
PLS: DA partial least squares discriminant analysis
PC: Phosphatidylcholine
PE: Phosphatidylethanolamine
PG: Phosphatidylglycerol
PI: Phosphatidylinositol
PS: Phosphatidylserine
Pro: Proline
Phe: Phenylalanine
RM: Right median lobe
RL: Right lateral lobe
ROS: Reactive oxygen species
RSD: Relative standard deviation
SM: Sphingomyelin
TG: Triacylglycerol
Tyr: Tyrosine
Trp: Tryptophan
Val: Valine
