# Supplementary material for: Metabolome profiling across liver lobes and metabolic shifts of the MASLD mice
Source: Genes Nutr. 2025 Apr 16;20:9. doi: 10.1186/s12263-025-00768-7 (PMC12001577; doi:10.1186/s12263-025-00768-7)
Supplement: Supplementary file 5 — Supplementary Material 5 [file 12263_2025_768_MOESM5_ESM.pdf]

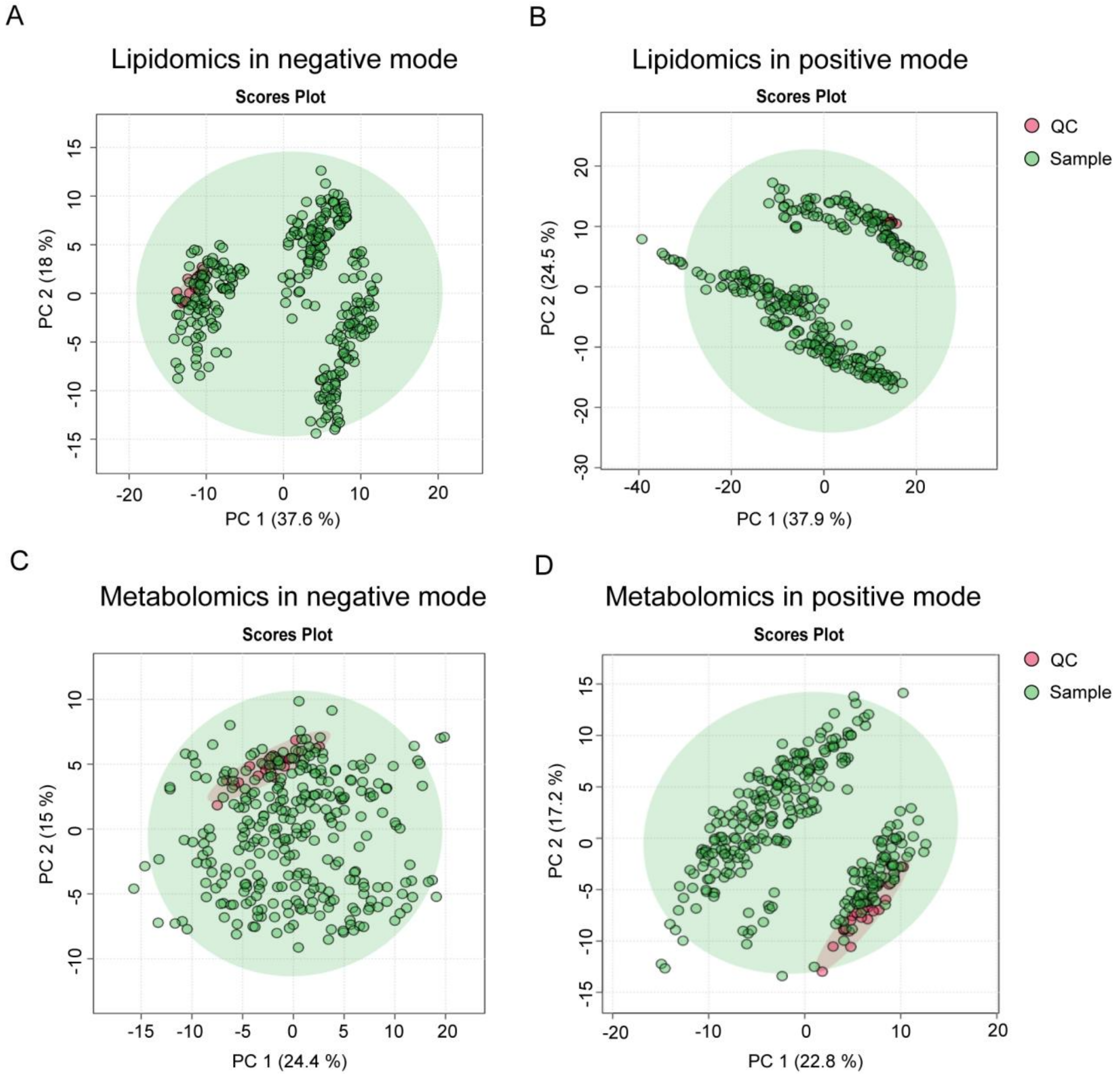

Supplementary Fig. 1 Quality control of the lipidomics and metabolomics analysis. (A) Two-dimensional PCA of lipidomics in negative mode. (B) Two-dimensional PCA of lipidomics in positive mode. (C) Two-dimensional PCA of metabolomics in negative mode. (D) Two-dimensional PCA of metabolomics in positive mode. QC samples clustered tightly on the score plot.

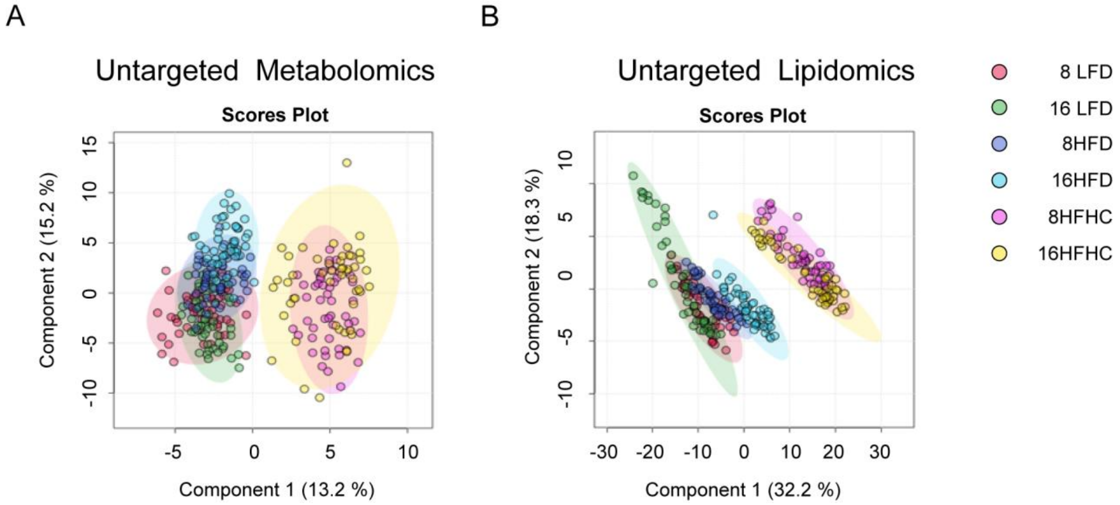

Supplementary Fig. 2 Results of PLS-DA for the of all liver samples.

(A) PLS-DA score plots of hepatic metabolities profiling among LFD, HFD and HFHC diet for different feeding periods (8 weeks or 16 weeks). (B) PLS-DA score plots of hepatic lipids profiling among LFD, HFD and HFHC diet for different feeding periods (8 weeks or 16 weeks). The x and y axes describe the first and second principal components, respectively, with the contributed ratios. n = 10 biologically independent samples. Each biospecimen included 5 liver lobe zonings (L1-L5).

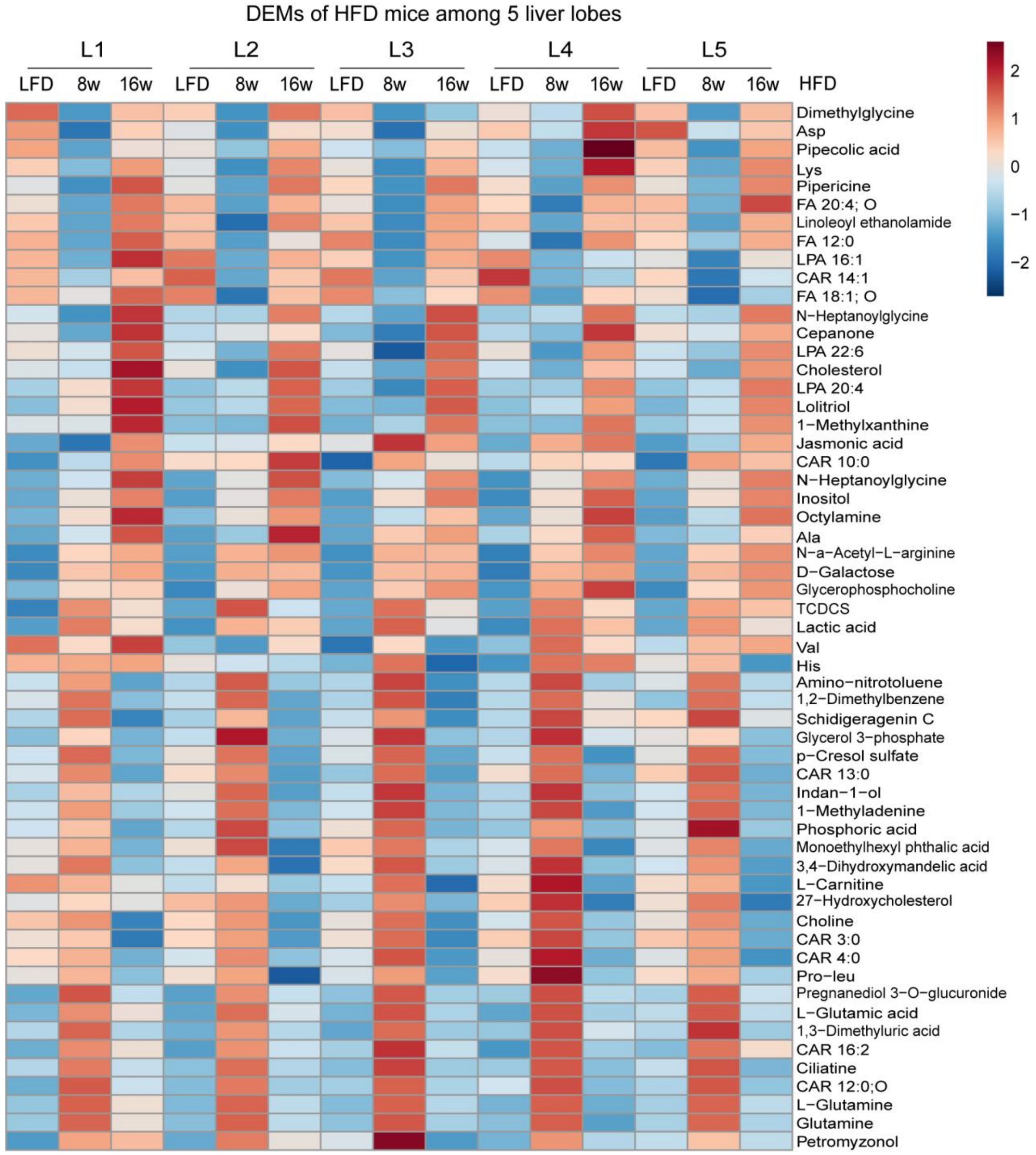

DEMs of HFD mice among 5 liver lobes
L1
L2
L3
L4
L5
LFD
8w
16w
HFD
2
1
0
−1
−2
Dimethylglycine
Asp
Pipecolic acid
Lys
Pipericine
FA 20:4; O
Linoleoyl ethanolamide
FA 12:0
LPA 16:1
CAR 14:1
FA 18:1; O
N−Heptanoylglycine
Cepanone
LPA 22:6
Cholesterol
LPA 20:4
Lolitriol
1−Methylxanthine
Jasmonic acid
CAR 10:0
N−Heptanoylglycine
Inositol
Octylamine
Ala
N−a−Acetyl−L−arginine
D−Galactose
Glycerophosphocholine
TCDCS
Lactic acid
Val
His
Amino−nitrotoluene
1,2−Dimethylbenzene
Schidigeragenin C
Glycerol 3−phosphate
p−Cresol sulfate
CAR 13:0
Indan−1−ol
1−Methyladenine
Phosphoric acid
Monoethylhexyl phthalic acid
3,4−Dihydroxymandelic acid
L−Carnitine
27−Hydroxycholesterol
Choline
CAR 3:0
CAR 4:0
Pro−leu
Pregnanediol 3−O−glucuronide
L−Glutamic acid
1,3−Dimethyluric acid
CAR 16:2
Ciliatine
CAR 12:0;O
L−Glutamine
Glutamine
Petromyzonol

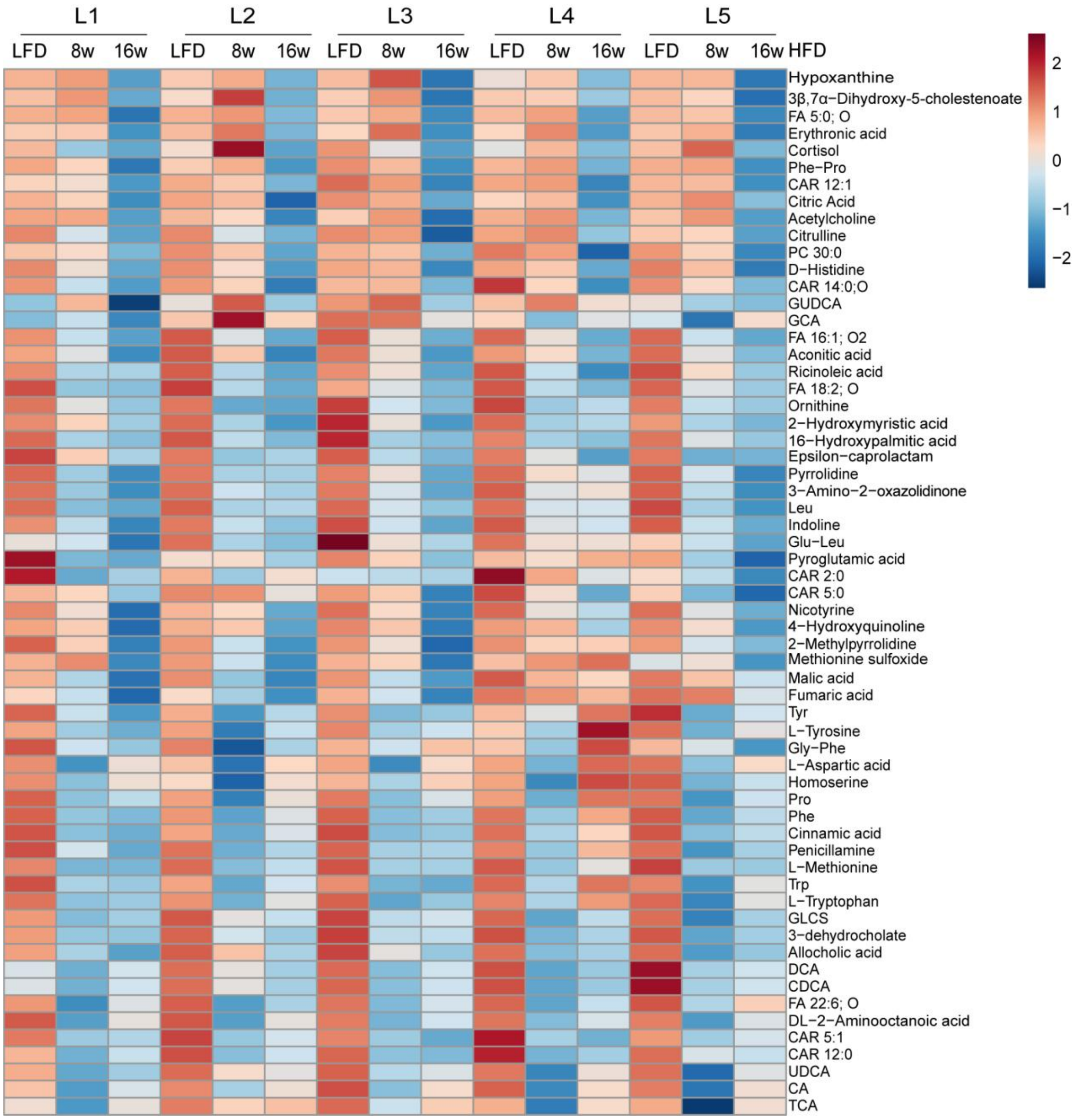

Supplementary Fig. 3 Heat maps of the differentially expressed metabolites (metabolome alone) among five liver lobes in mice fed HFD.

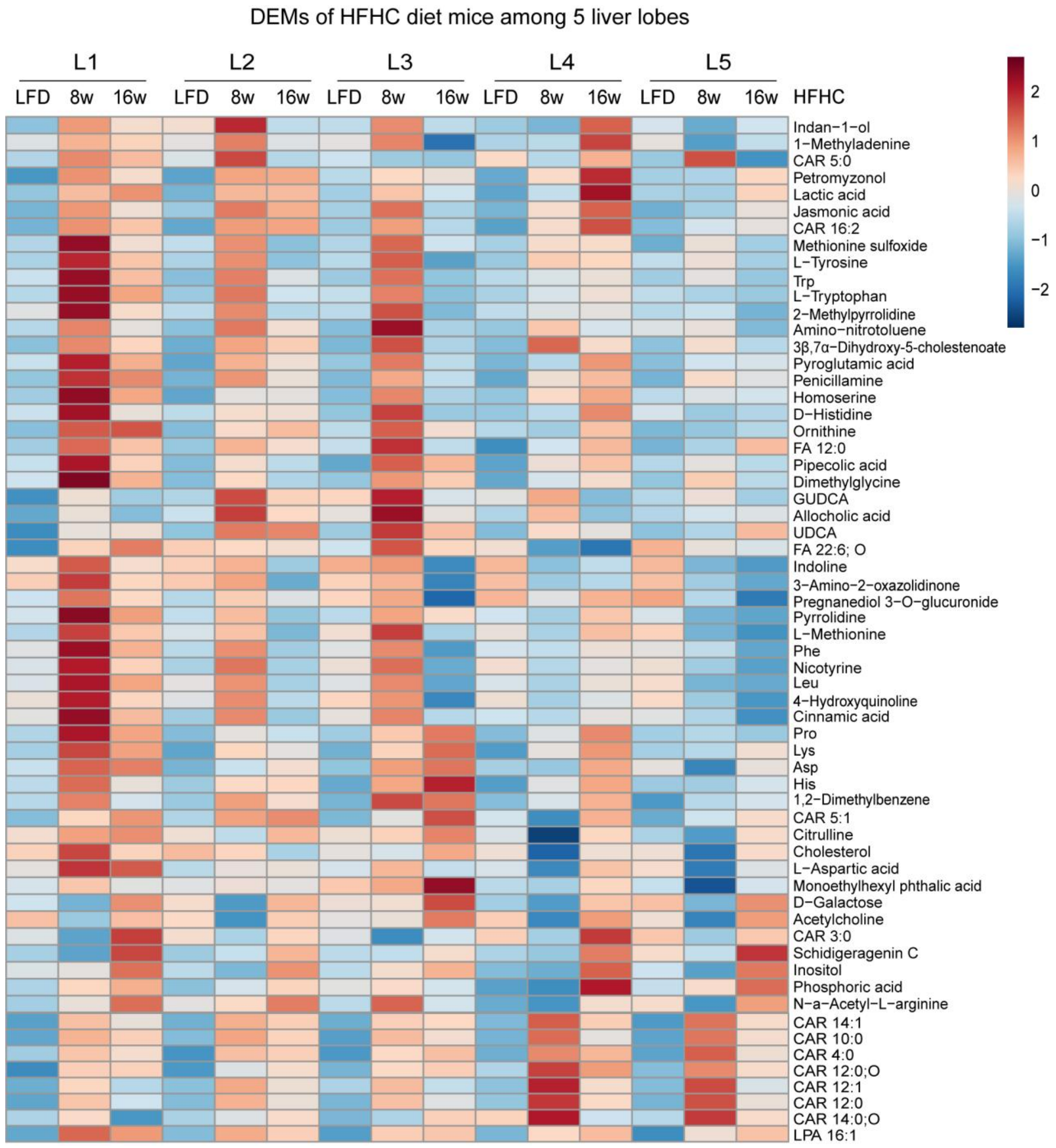
DEMs of HFHC diet mice among 5 liver lobes
L1
L2
L3
L4
L5
LFD
8w
16w
HFHC
Indan−1−ol
1−Methyladenine
CAR 5:0
Petromyzonol
Lactic acid
Jasmonic acid
CAR 16:2
Methionine sulfoxide
L−Tyrosine
Trp
L−Tryptophan
2−Methylpyrrolidine
Amino−nitrotoluene
3β,7α−Dihydroxy-5-cholestenoate
Pyroglutamic acid
Penicillamine
Homoserine
D−Histidine
Ornithine
FA 12:0
Pipecolic acid
Dimethylglycine
GUDCA
Allocholic acid
UDCA
FA 22:6; O
Indoline
3−Amino−2−oxazolidinone
Pregnanediol 3−O−glucuronide
Pyrrolidine
L−Methionine
Phe
Nicotyrine
Leu
4−Hydroxyquinoline
Cinnamic acid
Pro
Lys
Asp
His
1,2−Dimethylbenzene
CAR 5:1
Citrulline
Cholesterol
L−Aspartic acid
Monoethylhexyl phthalic acid
D−Galactose
Acetylcholine
CAR 3:0
Schidigeragenin C
Inositol
Phosphoric acid
N−a−Acetyl−L−arginine
CAR 14:1
CAR 10:0
CAR 4:0
CAR 12:0;O
CAR 12:1
CAR 12:0
CAR 14:0;O
LPA 16:1
2
1
0
−1
−2

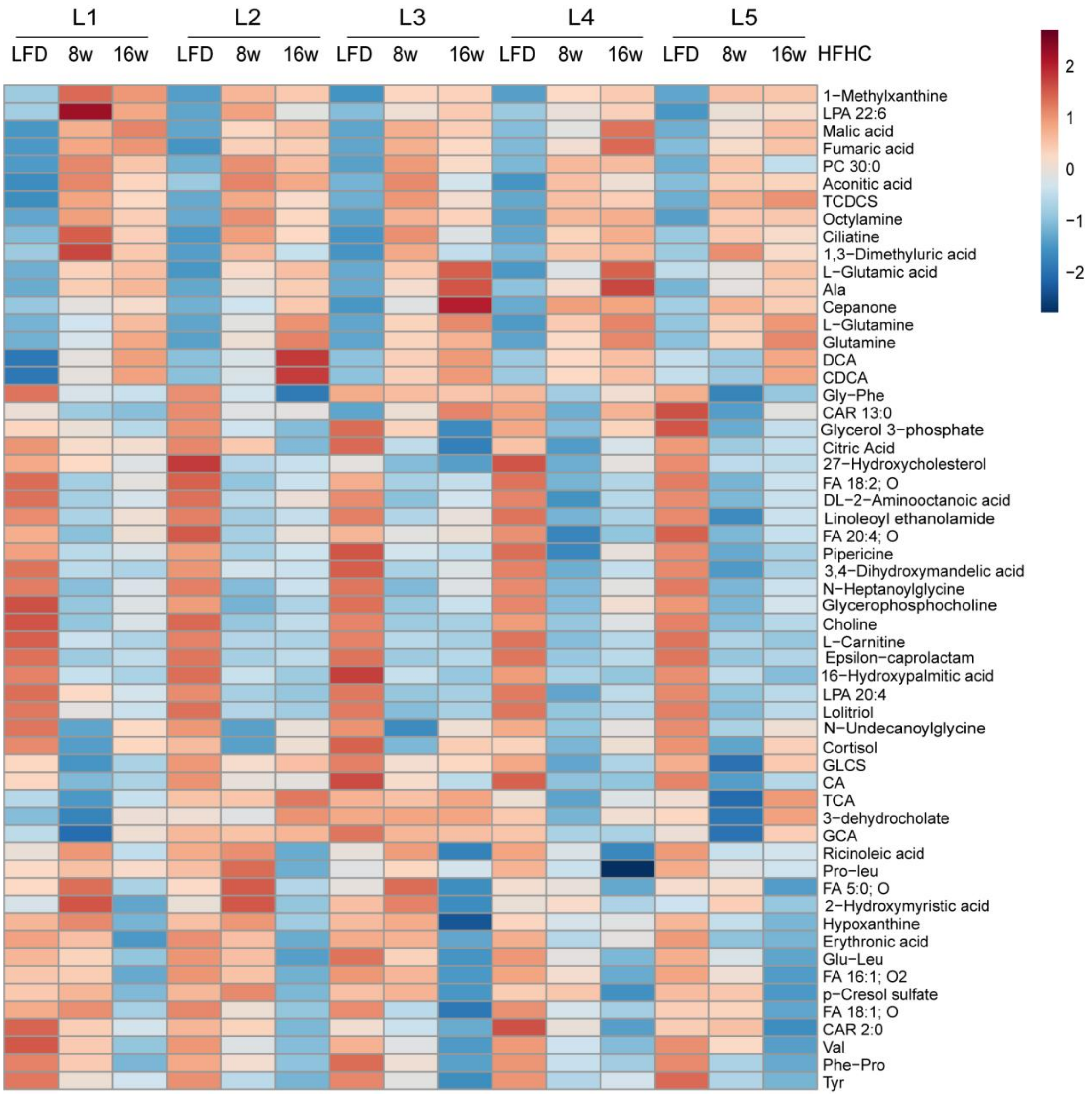

Supplementary Fig. 4 Heat maps of the differentially expressed metabolites (metabolome alone) among five liver lobes in mice fed HFHC diet.

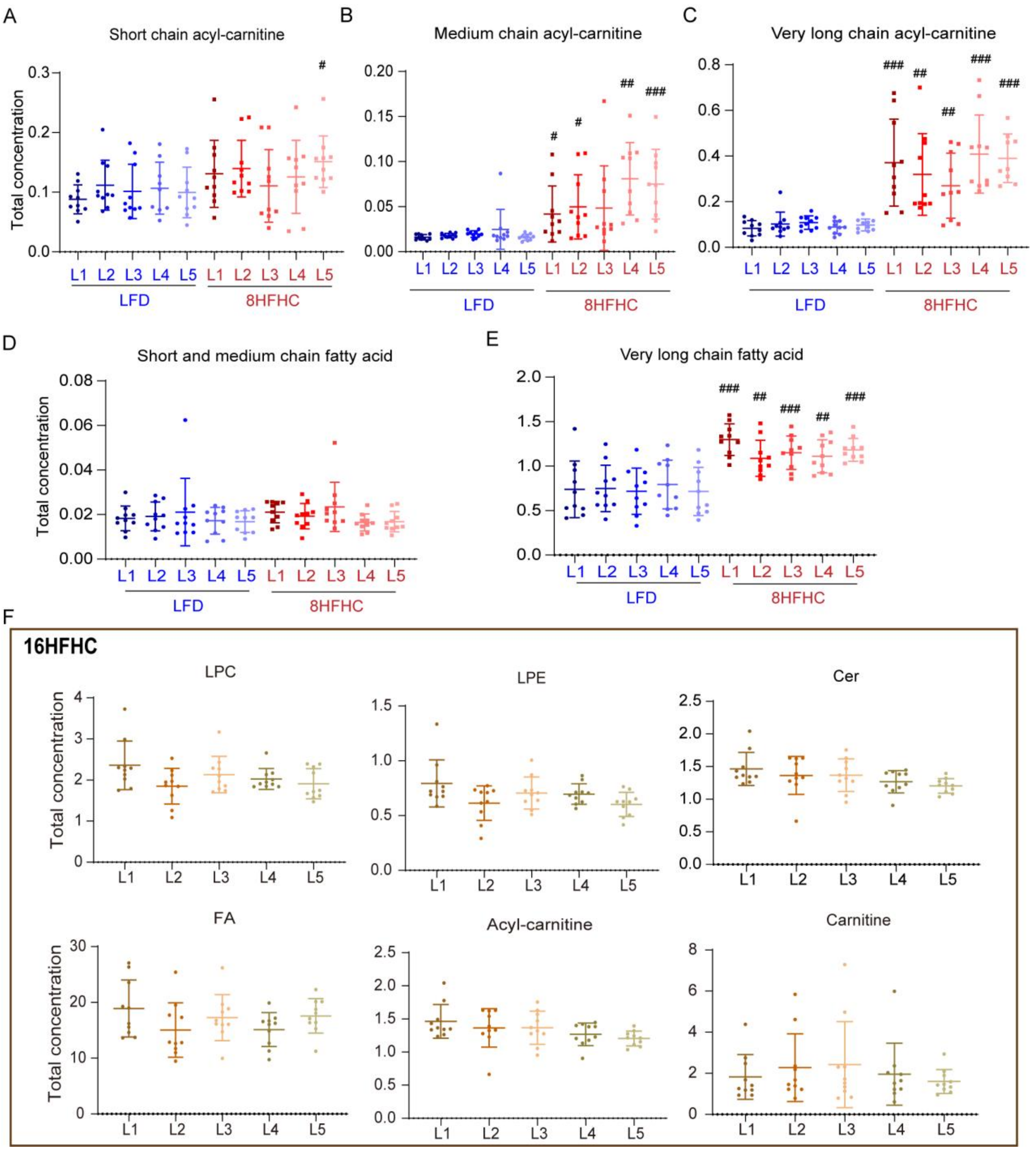

Supplementary Fig. 5 Changes of lipids among five liver lobes in MASLD progression stage.

(A-E) Changes in short chain acyl-carnitine (A), medium chain acyl-carnitine (B), very long chain acyl-carnitine (C), short and medium chain fatty acid (D), very long chain fatty acid (E) contents in different liver lobes of chow diet and 8 weeks HFHC diet mice. (F) Changes in LPC, LPE, Cer, FA, acyl-carnitine, L-carnitine contents in different liver lobes of 16 weeks HFHC diet mice. *indicates comparisons among liver lobe within the same diet. # indicates comparisons between two groups within the same liver lobe. */# P < 0.05, **/## P < 0.01, ***/### P < 0.001.
